# Changes in Dry State Hemoglobin over Time Do Not Increase the Potential for Oxidative DNA Damage in Dried Blood

**DOI:** 10.1371/journal.pone.0005110

**Published:** 2009-04-08

**Authors:** April Marrone, Jack Ballantyne

**Affiliations:** 1 Graduate Program in Chemistry, Department of Chemistry, University of Central Florida, Orlando, Florida, United States of America; 2 National Center for Forensic Science, Orlando, Florida, United States of America; Louisiana State University, United States of America

## Abstract

**Background:**

Hemoglobin (Hb) is the iron-containing oxygen transport protein present in the red blood cells of vertebrates. Ancient DNA and forensic scientists are particularly interested in Hb reactions in the dry state because both regularly encounter aged, dried bloodstains. The DNA in such stains may be oxidatively damaged and, in theory, may be deteriorated by the presence of Hb. To understand the nature of the oxidative systems potentially available to degrade DNA in the presence of dried Hb, we need to determine what molecular species Hb forms over time. These species will determine what type of iron (i.e. Fe^2+^/Fe^3+^/Fe^4+^) is available to participate in further chemical reactions. The availability of “free” iron will affect the ability of the system to undergo Fenton-type reactions which generate the highly reactive hydroxyl radical (OH•). The OH• can directly damage DNA.

**Methodology/Principal Findings:**

Oxygenated Hb (oxyHb) converts over time to oxidized Hb (metHb), but this happens more quickly in the dry state than in the hydrated state, as shown by monitoring stabilized oxyHb. In addition, dry state oxyHb converts into at least one other unknown species other than metHb. Although “free” iron was detectable as both Fe^2+^ and Fe^3+^ in dry and hydrated oxyHb and metHb, the amount of ions detected did not increase over time. There was no evidence that Hb becomes more prone to generating OH• as it ages in either the hydrated or dry states.

**Conclusions:**

The Hb molecule in the dried state undergoes oxidative changes and releases reactive Fe(II) cations. These changes, however, do not appear to increase the ability of Hb to act as a more aggressive Fenton reagent over time. Nevertheless, the presence of Hb in the vicinity of DNA in dried bloodstains creates the opportunity for OH•-induced oxidative damage to the deoxyribose sugar and the DNA nucleobases.

## Introduction

Hemoglobin (Hb) is the iron-containing oxygen transport protein present in the red blood cells of vertebrates (Hemoglobin A for humans). Oxygenated Hb (oxyHb) is a low-spin ferrous compound that gives blood its characteristic red color. OxyHb is easily oxidized under the influence of external oxidants to methemoglobin (metHb), which is a high-spin ferric protein that can no longer bind elemental oxygen. Over time, the high-spin ferric compound can convert to various low-spin ferric forms called hemichromes (scheme 1). Hemichromes are formed through changes of protein conformation so that atoms endogenous to the protein become bonded to the iron as the sixth ligand. Because Hb is a major component of blood (a body fluid often subjected to forensic DNA analysis), it is important to understand molecular transformations of the Hb molecule that could lead to possible oxidative damage to the other components of a blood stain, particularly DNA.

### Scheme 1

In this notation, the superscript denotes the number of d electrons in the iron atom and the subscript is the total spin of the iron atom.
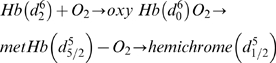



For oxidative damage to occur, oxidizing agents must be available that can interact with biomolecules such as DNA. One of the most damaging of these agents is the hydroxyl radical (OH•), which can be produced during a biological Fenton type reaction catalyzed most likely by ‘free’ iron [1]–[4]. Native Hb contains four heme groups, each of which contains an iron center. It is unlikely that the iron complexed with Hb itself produces OH• capable of interacting with other biomolecules; such radicals produced at the iron center would have to travel through the protein into free solution to react. Thus, the formation of OH• in this manner would most likely lead to oxidative damage of the parent Hb molecule.

To our knowledge, there have been no previous studies conducted on how Hb in the dried state affects the oxidation of other cellular components, specifically DNA. It can be hypothesized, based on previous research on the destructive role of ionic iron *in vivo*, that oxidative damage could be exacerbated by the presence of Hb and its potential release of ‘free’ iron. The handling of ‘free’ iron inside the living body is carefully regulated via metabolic pathways which help keep the formation of cytotoxic OH• under control [5], [6]. An overload of ionic iron is correlated with DNA oxidative damage.[7] These metabolic pathways would not be functional in dried bloodstains. However, in the forensic context some damage to DNA in bloodstains is expected and is likely to be more pronounced in older samples.[8], [9] Upon recovery of a dried bloodstain from a crime scene, the potential role that Hb can play in subsequent damage to the sample can be inferred by the present study.

In this work, we have sought to characterize the molecular species formed by Hb maintained in the dry state at ambient temperatures and humidity over a period of time. First we determined the presence and/or formation of Hb isoforms because these species will determine what type of iron (i.e. Fe^2+^/Fe^3+^/Fe^4+^) is available to participate in further chemical reactions. We also determined whether, and to what extent, free iron is released from Hb because this together with its oxidation status will affect the ability of the system to undergo Fenton type reactions. Finally, the ability of Hb to inflict oxidative damage on a deoxyribose substrate, presumably through the formation of OH•, was measured as a function of the age of the dried Hb.

## Results

### Oxidation of Human Hemoglobin

The dry state Hb samples used in the initial experiments were in their oxidized form (oxyHb) according to their measured UV spectra. Initially, ferrous-stabilized Hb was used to measure the relative rates of oxyHb oxidation between hydrated and dry state Hb. Without the stabilization process, the Hb would have been primarily in the metHb oxidized ferric form upon receipt in the lab from the commercial vendor due to the inherent proclivity of the metalloprotein to undergo ferrous ion oxidation over time. OxyHb samples in the dried and hydrated states were maintained at ambient temperature (22.0±0.4°C) and relative humidity (54±7%) in the dark for varying periods up to 3 months (2200 hours). Oxidation product formation was monitored by visible region absorption spectrophotometry. The concentrations of oxyHb, metHb, and presumed hemichrome were measured as a function of time.

**Figure 1:**
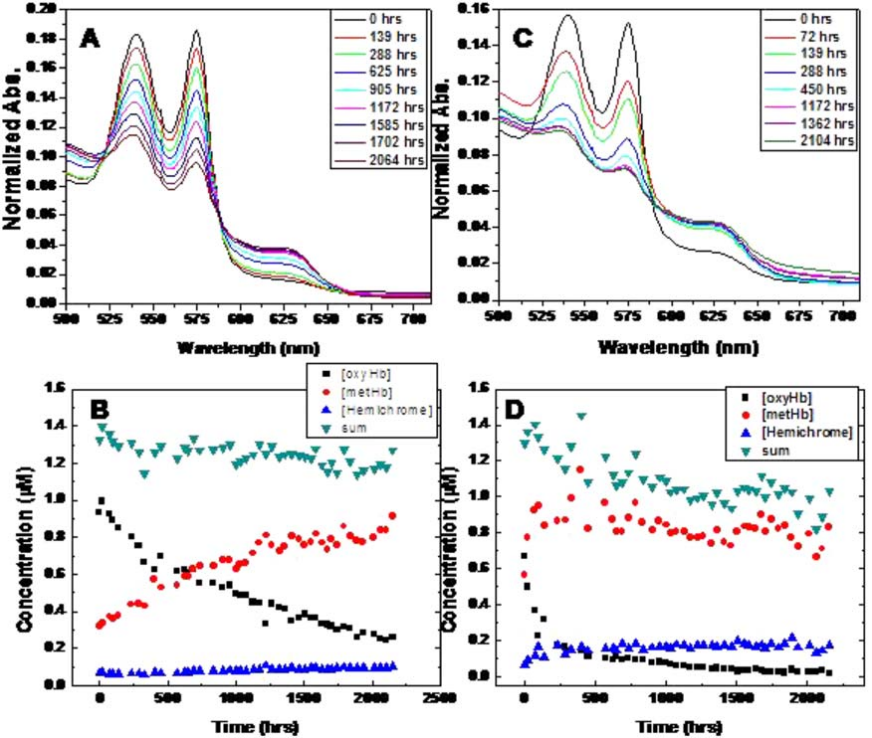
All data comprise an average of three samples. (A) Spectra of hydrated Hb at various time periods where it is evident that the oxyHb is oxidizing to primarily metHb. (B) Concentration of oxyHb (▪), metHb (•), and hemichromes (▴) from hydrated Hb incubated over a 2200 hour time period in ambient conditions. (C) Spectra of dry Hb at various time periods where it is evident that the oxyHb is oxidizing to not only metHb, but what is suspected to be hemichromes. (D) Concentrations of oxyHb (▪), metHb (•), and hemichromes (▴) from dry Hb incubated over a 2200 hour time period in ambient conditions.

**Figure 2:**
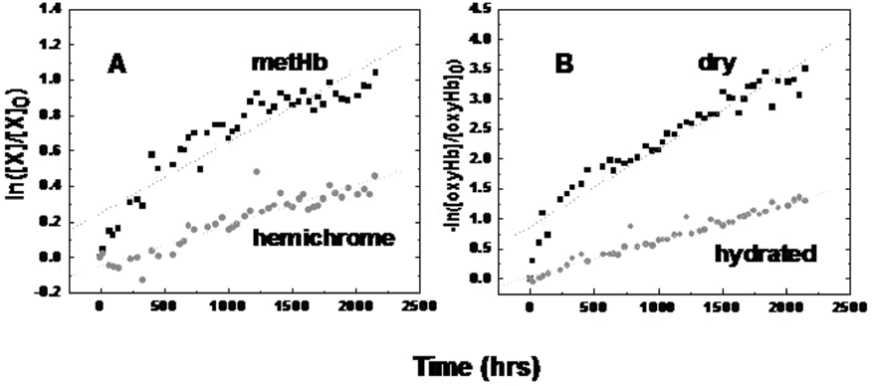
All data are an average of three samples. (A) Rate determination for the formation of metHb (▪) and hemichromes (•) from hydrated samples. The rates are k = 1.11±0.07×10^−7^ s^−1^ (R = 0.91735) and k = 6.29±0.4×10^−8^ s^−1^ (R = 0.92667) respectively. (B) Rate determination for the oxidation of oxyHb by plotting −ln([oxyHb]/[oxyHb]_0_) vs. time for both dry (▪) and hydrated (•) samples. The rates are k = 3.58±0.17×10^−7^ s^−1^ (R = 0.95618) and k = 1.69±0.06×10^−7^ s^−1^ (R = 0.97785) respectively.

The spectra of hydrated oxyHb over time (**Figure 1A**) indicated the likely presence of only two major species because two isosbestic points at 524 nm and 590 nm were identified. The spectra shown are an average of three separate samples incubated over the same time period and might account for the minor variation of spectra around the 524 nm isosbestic point. However, to further investigate the number of Hb species formed, we compared the rates of formation of solely metHb or the formation of metHb and hemichromes as a second product with the rate of degradation of oxyHb. If only two species were present (viz. oxyHb and metHb), then oxyHb appeared to degrade at a rate of 1.53±0.04×10^−7^ s^−1^ and metHb formed at a rate of 1.13±0.07×10^−7^ s^−1^. If there were a strict one to one relationship between reactant and product, the two rates should be the same. However, calculation of the t-statistic indicates that the rates are significantly different (t = 33.3) at the 95% confidence level. In **Figure 2A**, the rate of oxyHb loss over time was determined under the assumption that hemichromes were also being formed. In this scenario, oxyHb degraded at a rate of 1.69±0.06×10^−7^ s^−1^ in the hydrated state. MetHb formed at a rate of 1.11±0.08×10^−7^ s^−1^ and hemichrome formed at a rate of 6.29±0.40×10^−8^ s^−1^. The combined rate for both metHb and hemichrome formation was 1.74±0.08×10^−7^ s^−1^, which should be the same as that of oxyHb degradation if both products were being formed. Although the t-statistic indicated that the rates were different, the value (t = 3.35) was ten times smaller than the one calculated without assuming the presence of product species other than metHb. The sums of the estimated concentrations of the reactant (oxyHb) and proposed products (metHb and hemichromes) appeared to be reasonably constant over time (**Figure 1B**), although a slight decrease was discernible. It is possible that the latter observation might indicate the presence of another minor as-yet-unidentified product.

In contrast to the hydrated state, dry state oxyHb lacked the two isosbestic points in the time evolved-spectra (**Figure 1C**). Thus, more than two species were present with the initial hypothesis being that, like hydrated Hb, hemichromes were being formed in addition to metHb but in larger quantities than with the hydrated samples. This hypothesis is supported by the approximately 2-fold increased rate of oxidation measured for dry state oxyHb (k = 3.58±0.17×10^−7^ s^−1^) compared to the hydrated state (**Figure 2B**). Although a degradation rate could be determined for dry state oxyHb, the formation of metHb and hemichrome over time did not appear to be a first order reaction. The sum of the three species (i.e. the reactant, oxyHb and the products, metHb and hemichromes) did not remain constant over time (**Figure 1D**) and is consistent with the presence of a fourth species. The putative fourth species could be a denatured Hb derivative that is not detectable by the methods employed here. Considering the nature of the Hb metalloprotein, other potential species that might be formed include ferrylHb and choleglobin. FerryHb is an Fe(IV) complex formed from ferrous hemoglobin and H_2_O_2_ whereas choleglobin is denatured hemoglobin in which the porphyrin ring has been hydroxylated or broken open. The ferrylHb species was ruled out because: (a) there was no exposure to H_2_O_2_, nor were there any environmental conditions that would lead to such exposure; and, (b) the spectra do not show evidence of its existence, primarily by the lack of spectral broadening where the 577 nm shoulder drops off steeply around 585 nm. There was also no spectral evidence of the presence of choleglobin due to the lack of increased absorption at 700 nm as is characteristic of the species [10].

**Figure 3:**
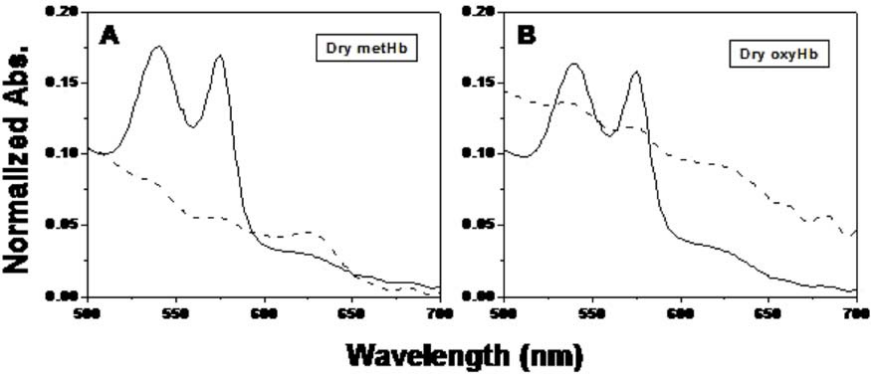
Spectra of dry metHb (A) and dry oxyHb (B) prior to (----) and after (—) reduction with sodium dithionite. All spectra were measured after ∼1000 hours.

After oxyHb oxidation to metHb had taken place, there was no evidence in either hydrated or dry state for further significant structural transformations that would cause changes in the absorption spectrum (data not shown). After reduction with sodium dithionite, dry state metH**b** and dry state oxyHb (**Figure 3A and 3B**); maintained at ambient temperature and humidity for 1000 hours were converted to Hb primarily in the oxyHb form. Hydrated-state oxyHb and metHb samples similarly treated were also converted to oxyHb (data not shown). The characteristic spectrum of hemochrome was not detected in any sample after reduction. Thus, we concluded that the formation of hemochromes was not responsible for the additional unknown species in degraded oxyHb.

### Release of Iron Cations from Hb

Human Hb that was primarily in the form of metHb was incubated in the dry and hydrated states over a 1000 hour time period, and the free iron released from the Hb was measured at various time intervals. Fe(II) was measured directly by interaction with ferrozine whereas Fe(II) plus Fe(III) was determined after reduction with ascorbic acid. The amount of Fe(III) is thus indicated by the differences in the two response curves.

**Figure 4:**
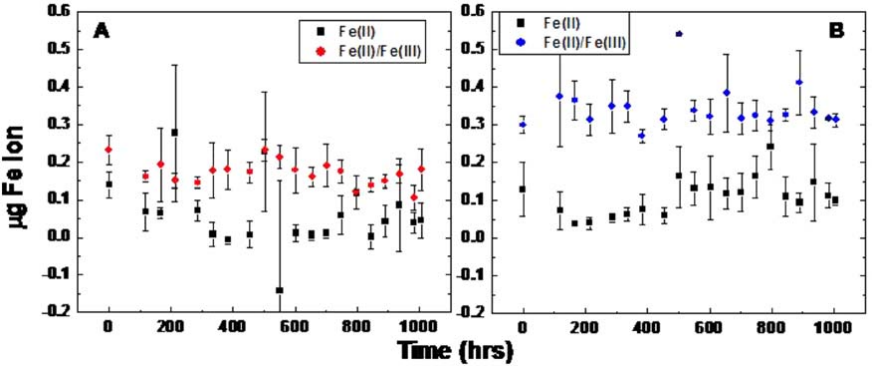
Free iron detected. (A) Free iron present in hydrated Hb, ▪ = Fe^2+^, • = Fe^2+^+Fe^3+^. (B) Free iron present in dry state Hb, ▪ = Fe^2+^, • = Fe^2+^+Fe^3+^.

Free Fe(III) was detected in both the hydrated (**Figure 4A**) and dry (**Figure 4B**) state oxyHb samples. This was expected because it was known that the oxyHb had oxidized into primarily metHb at the time the measurements were performed. Though Fe(III) appeared to be the dominant form of free iron, some Fe(II) was present. We hypothesize that Fe(II) was released while the protein was still in its oxyHb form, but as the protein was oxidized to metHb, the iron that continued to be released was in the +3 oxidation state. What was surprising was that over time the Hb samples did not continue to release free iron in either state, as is evidenced by the lack of an increase in free iron. The dry samples did exhibit an insignificant increase in the release of free Fe(II), perhaps due to some configuration that the protein takes in the dry state that leads to more favorable release of the ion during the dehydration process. Overall, these results imply that dried bloodstains may provide reactive free Fe(II) that can engage in a Fenton type reaction, but that the age of a bloodstain may not be a significant factor in its ability to do so.

### Hydroxyl Radical Detection

**Figure 5:**
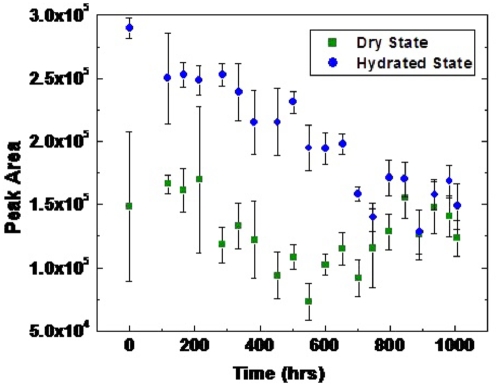
MetHb incubated over time at 21.9±0.1°C and 61.3±1.0 relative humidity ((▪) dry state, (•) hydrated state). The samples were reacted with deoxyribose and then thiobarbituric acid to detect oxidative damage to the deoxyribose. The absorption measured at 528 nm is given after blank subtraction. Measurements are the average of three different samples incubated at the same time point.

Hb primarily in the form of metHb was incubated over time at ambient temperature (21.9±0.1°C) and relative humidity (61.3±1.0%). The samples were reacted with deoxyribose and then thiobarbituric acid to detect oxidative damage to the deoxyribose. Oxidative damage due to OH• attack of deoxyribose was considered to have occurred if the absorption of the pink chromogen after incubation with metHb was greater than incubation in the absence of metHb. The peak area obtained by measuring the spectra at 532 nm was used to determine relative amounts of damage to each sample after blank subtraction. The hydrated state metHb caused the most hydroxyl radical damage at the initial time point before being left to sit in ambient conditions and the reactivity decreased over time (**Figure 5**). The dry state metHb displayed much less oxidative ability and the reactivity did not change noticeably during the time period (**Figure 5**).

## Discussion

The data presented in this study indicate that dry state Hb undergoes more rapid oxidation than that in a hydrated state. In both states, however, the resulting product is Hb in which the Fe center has been oxidized to Fe(III). At least one other species is believed to be present as the result of the oxidation of oxyHb in both the hydrated and dry states (particularly the dry state), but its identity has eluded the experimental schema employed here. In the hydrated state at neutral pH, the oxidation of oxyHb to metHb and the reduction of metHb to oxyHb have approximately the same rate constant [11]. However, over extended periods such as was experienced by the samples here, this equilibrium eventually ceases to hold and the metHb species becomes more prevalent. It is possible that dry state oxyHb forms metHb more rapidly than does the hydrated form due to the lack of dynamic equilibrium that the hydrated state offers.

Formation of OH• requires the presence of iron salts, specifically Fe(II). Other transition metals or iron-protein complexes including Hb are unable to catalyze the reaction [12]. It is believed that the most likely route for oxidative damage to DNA caused by a Fenton type reaction involving Fe(II) is for the ferrous ion to bind to the deoxyribose molecule with a certain affinity and then induce site-specific damage. This hypothesis is supported by prior studies carried out by Gutteridge [13], [14] where it was noted in such systems that the reaction of the carbohydrate with OH• was poorly inhibited by most OH• scavengers. In addition to experimental evidence, a theoretical analysis of the thermodynamics of a “Fenton type” reaction offers evidence for an inner-shell or bridged reaction mechanism [1].

It has been previously reported that OH• can be generated in a reaction that is independent of O_2_^•−^ by the addition of Fe(II) salts alone [12], [15] (Reactions 1,2 and 3 below). This was determined by Halliwell and Gutteridge by the inability of superoxide dismutase to prevent deoxyribose degradation. However, catalase did prevent damage indicating that H_2_O_2_ is involved in the reaction, despite it not being added to the reaction mixture [15]. Ferric ion was incapable of degrading the deoxyribose substrate without the addition of a superoxide-generating system (xanthine/xanthine oxidase). The O_2_•^−^ most likely reduces Fe(III) to Fe(II) (Reaction 4) which then allows for reaction 1 to occur [16], [17]. The net reaction of reactions 3 and 4 is Reaction 5 (Haber Weiss reaction). The Haber Weiss reaction is the underlying phenomenon that is believed to be the principal source of OH• in biochemical systems [12]. Thus, there is protection offered to samples due to the inherent nature of iron to oxidize to the ferric state.
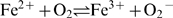
(1)

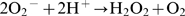
(2)

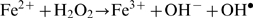
(3)

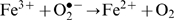
(4)

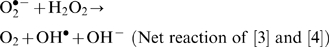
(5)


OxyHb and metHb have been shown previously to form hydroxyl radicals in the presence of hydrogen peroxide [18]. Although studies were not carried out here with H_2_O_2_, it was noted by Halliwell and Gutteridge [19] that metHb does degrade deoxyribose, and this degradation was increased when ascorbic acid was added to the reaction mixture. The ascorbic acid would reduce any free Fe(III) and allow for a better catalyst for the formation of OH•.

Although it is believed that iron bound to Hb will not produce free OH• in solution, ‘free’ iron can do so. It is possible to degrade Hb using peroxides to release the metal center [20], allowing for Fenton type chemistry. It was shown here that, in the absence of H_2_O_2_ or any other organic hydroperoxides, small amounts of free iron can be released from the Hb molecule. The small amounts of unbound iron are sufficient to degrade deoxyribose to an extent that exceeds that of deoxyribose heated in the absence of Hb. It was also observed that dry state metHb did not degrade deoxyribose as extensively as hydrated state metHb did. This was unexpected as the amount of ‘free’ Fe(II) was measured to be slightly greater in dry state metHb. Overall, the age of metHb samples did not influence their ability to generate OH• and cause oxidative damage. Though it is apparent that Fenton type chemistry is likely to occur in a Hb-containing system such as a dried bloodstain, the damaging capabilities of such a system do not appear to increase as the age of the system increases - at least with the relatively mild laboratory conditions studied here. Further studies would have to be performed to determine whether the same holds for samples maintained under conditions representative of more extreme climatic conditions. Additionally, due to the presence of all cellular components in bloodstains (including pools of non-Hb sources of ‘free’ iron), the dynamics of the oxidative damage process may differ from the one studied here in isolation.

Based on the findings here, the oxidative damage that would be incurred by a dried stain sample containing the Hb molecule would most likely occur prior to the receipt of such a sample by the analyst. Therefore, subsequent storage of such samples should not result in further damage induced by Hb-derived hydroxyl radicals.

## Materials and Methods

### Sample Preparation and Analysis

All dry state samples were created by vacuum centrifugation and then maintained at room temperature in the dark at ambient temperature and humidity conditions (22.0±0.4°C, 54±7% relative humidity) for varying periods up to approximately three months. Samples were removed from the ambient environment at various time points in triplicate and frozen. Dry state samples were prepared for analysis by re-hydrating in a total volume of 50 µl of de-ionized water (same volume as hydrated samples) unless otherwise stated.

All spectra were measured using a UV6000 diode array detector (ThermoElectron, Waltham, MA, USA), equipped with a 5 cm light-path flow cell. The detector was in line with a SpectraSystem P200 pump and autosampler which supplied buffer (0.5 mM Tris HCl, 0.1 mM EDTA) at a flow rate of 1 ml/min through the system. Data were analyzed using the XCalibur® software package provided by the manufacturer.

Human A_0_ stabilized Hb, human Hb mainly in the form of metHb, and 2-deoxy-D-ribose (deoxyribose) were purchased from Sigma Aldrich (St. Lois, MO, USA). Ammonium acetate, ammonium Fe(II) sulfate hexahydrate, ascorbic acid, ferrozine, neocuproin, thiobarbituric acid (TBA), trichloroacetic acid (TAA), and sodium dithionite were purchased from Fisher Scientific (Pittsburgh, PA, USA).

### Oxidation of Human Haemoglobin

A stock solution of ferrous-stabilized human A_0_ Hb was made by diluting 5.0 g of product in 10 ml of 0.5 mM Tris HCl and 0.1 mM EDTA. The stabilized Hb product had less than 15% metHb and was primarily in the oxyHb form as verified by spectral analysis. The means by which this product is stabilized is proprietary to the supplier, although ficoll and sucrose are present. Therefore, measuring the mass of the product does not allow for determination of the amount of Hb. The determination of Hb concentration in samples was determined using molar absorbtivities (Table 1) provided by Winterbourn [11]. Individual samples were made with 50 µl aliquots of the stock solution. A 20 µg/µl stock solution of human Hb which was primarily in the form of metHb (both stated by the manufacturer and confirmed by spectral analysis) was used to make individual 100 µl samples containing 2 mg of Hb.

**Table 1:** Millimolar absorption coefficients of Haemoglobin derivatives.†

	Wavelength (nm)		
Derivative	560	577	630
oxyHb [41]	8.6	15.0	0.17
methHb [42]	4.30	4.45	3.63
ferrylHb [43]	14.1	3.9	3.0
Hemichrome	8.6	6.8	0.92

† All values are expressed per heme group.

After incubation and re-hydration where necessary, oxyHb samples were analyzed without further preparation. Three distinct species were speculated to be prevalen:; oxyHb; metHb; and, hemichromes. FerrylHb was also considered to be a possible product. Using the millimolar extinction coefficients of the Hb derivatives, equation sets 1 and 2 were used to determine the amount (µM) of each species present considering the 5 cm path length of the instrument.
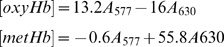
(1)

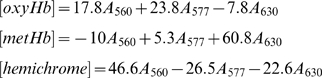
(2)


Dry metHb samples were re-hydrated with 1.1 ml of water. An additional 1.0 ml of water was added to the hydrated samples to bring their total volume to 1.1 ml as well. From each sample, 13.75 µl was taken and diluted to 100 µl forming 250 ppm solutions for which absorption spectra were measured from 200–800 nm.

To detect the presence of hemichromes against the background of oxyHb and metHb, samples were reduced and their spectra were measured to look for characteristic peaks at 529 and 558 nm as indicative of hemochromes [21]. Solid sodium dithionite was added directly to the Hb solutions and allowed to react at room temperature for 30 minutes. Samples were then injected into dialysis cassettes (Fisher Scientific, USA) and dialyzed against a 0.1 M phosphate buffer (pH 7.0) overnight.

### Release of Iron Cations from Hb

Free Fe(II) ions was determined using the ferrozine method of Carter [22]. Ferrozine, a disodium salt of 3-(2-pyridyl)-5,6-bis-(4-phenylsulfonic)acid)-1,2,4-triazine [23], forms a stable magenta-colored compound with the ferrous ion in a 3∶1 ratio and has an absorption peak at 562 nm. Neocuproine forms a complex with copper(I) and copper(II) that can be used in conjunction with ferrozine to keep copper ions from interfering with the ferrozine method [24], [25], and thus was also incorporated into the reaction. Human Hb that was primarily in the form of metHb was incubated in dry and hydrated (20 µg/µl) states over a 42 day time period. Samples were then diluted to a concentration of 1.82 µg/µl and two aliquots each of 500 µl were taken from each sample. One of the aliquots was then reduced by the addition of 500 µl reducing agent (ascorbic acid) and allowed to sit at room temperature for five minutes. This will result in the reduction of any Fe(III) present to Fe(II). The other aliquot was not reduced and this sample represents the amount of Fe(II) present. The Hb was then removed from both aliquots by the addition of 500 µl protein precipitant and centrifuged at 3000 rpm for five minutes. A volume of 1 ml was then removed from the reduced samples and to this was added 400 µl buffer and 100 µl ferroin reagent. A volume of 500 µl was removed from the non-reduced samples and to this was added 200 µl buffer and 100 µl ferroin reagent. The magenta-colored chromophore formed within five minutes and the stability of the complex was sufficient enough to allow for spectrophotometric analysis at 562 nm up to at least one day after formation. The difference in the response curves for the non-reduced (Fe(II)) and reduced (Fe(II)+Fe(III)) samples represents the amount of Fe(III) present.

### Reagents

#### Reducing Agent

A 0.2% ascorbic acid in 0.2 N hydrochloric acid solution was made by dissolving 20 mg ascorbic acid in a 100 ml volumetric flask with 1.67 ml of concentrated hydrochloric acid and deoinized water. The solution was stored at 4°C for ≤3 days prior to use.

#### Protein Precipitant

An 11.3% trichloroacetic acid (TAA) solution was made by dissolving 11.3 g of TAA in a 100 ml volumetric flask with deionized water.

#### Buffer Solution

A 10% ammonium acetate buffer solution was made by diluting 10 mg of ammonium acetate with deionized water in a 100 ml volumetric flask.

#### Ferroin Color Reagent

The ferrion color reagent consists of both ferrozine (0.3% w/v) and neocuproine (0.3% w/v) in aqueous solution. 300 mg of ferrozine and 300 mg of neocuproine were dissolved in deionized water using a few drops of concentrated hydrochloric acid to aid is dissolution and diluted to 100 ml in a volumetric flask.

#### Standard Iron Solutions

A standard iron solution was made by dissolving 702 mg Fe(NH_4_)_2_SO_4_•6H_2_O with 0.5 ml concentrated sulfuric acid into a 1 liter flask. Standards were then made consisting of 0, 0.195, 0.476, 0.909, 1.15, and 1.45 µg/ml Fe(II). These standards were used to construct a calibration curve for Fe(II) determination in samples.

### Hydroxyl Radical Detection

A thiobarbituric acid (TBA) assay for hydroxyl radical (OH•) detection was used that is based upon the detection of hydroxyl radical attack on, and degradation of, the sugar deoxyribose (2-deoxy-D-ribose) [2], [3], [12], [13], [15]–[17], [19], [20], [26]–[39]. When the resulting degradation product is heated under acidic conditions, malondialdehyde (MDA) is formed and that is detected by its ability to react with thiobarbituric acid (TBA) to form a pink chromogen. This assay has a high degree of specificity for OH• detection because other oxidizing species such as peroxyl and alkoxyl radicals do not release TBA reactive materials from deoxyribose [35].

**Figure 6:**
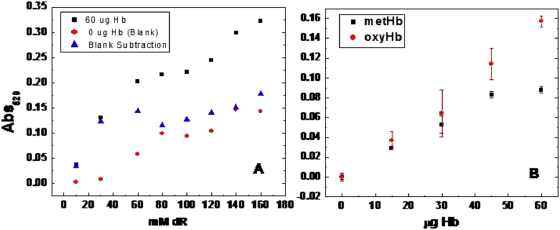
Thiobarbituric Acid (TBA) test for hydroxyl radical damaged deoxyribose. (A) 60 µg Hb incubated with various concentrations of deoxyribose. (B) 100 mM deoxyribose incubated with various amounts of ferrous (•) and ferric (▪) Hb. All samples were incubated with TBA at 95°C for 15 minutes to create a pink chromagen and spectral analysis performed at 532 nm.

Preliminary studies of the TBA reaction were conducted to optimize deoxyribose concentration and incubation parameters. **Figure 6** shows the absorbance measurement at 532 nm using 60 µg Hb and varying the concentration of deoxyribose at 37°C. The assay concept was to reach a point where the deoxyribose is not continuing to absorb radiation regardless of the concentration. It can be seen that the absorbtion increases linearly up until approximately 60 mM, where the absorption becomes somewhat constant until approximately 100 mM. Above 100 mM, the absorption increases again linearly. This trend was not only noted for samples containing 60 µg Hb, but for the blank samples containing no Hb. The absorbance of the blank samples containing only deoxyribose treated with TBA increases with deoxyribose concentration, especially at concentration larger than 100 mM; therefore, the curve will always increase slightly as the concentration of deoxyribose increases without degradation by Hb. The same figure shows the absorbance curve holding the concentration of deoxyribose steady at 100 mM and increasing the amount of Hb. At 100 mM, the increase is linearly responsive to Hb concentration. Based on these results we decided to use a 100 mM concentration of deoxyribose in the assay.

**Figure 7:**
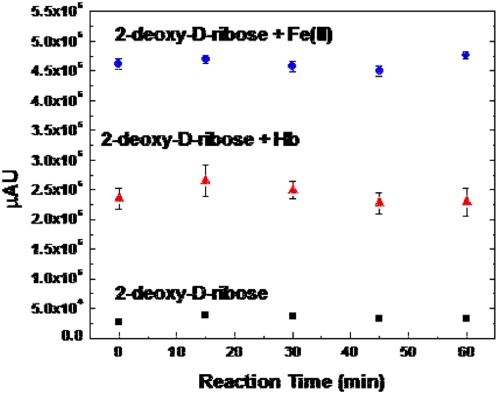
Reaction of 20 mM deoxyribose with Fe(II) and ferrous Haemoglobin (Hb) incubated at 37°C over time periods of up to one hour. The sugar is degraded on exposure to hydroxyl radicals. The reaction mixure is heated under acidic conditions using TAA to form malondialdehyde (MDA) which reacts with TBA to form a pink chromogen. The absorption was measured at 532 nm after incubation of deoxyribose (▪), with 1.66 µg Fe(II) (•), and with 35 µg Hb (▴).

The incubation period for reaction with deoxyribose was evaluated using an Fe(II) standard and oxyHb for up to 1 hour and testing samples every 15 minutes (**Figure 7**). From this study it was determined that there was not much difference in incubating with deoxyribose over a period of 15 minutes or for 1 hour. It was decided to use the 15 minute period so as not to reach a point where the maximum amount of decomposition occurs regardless of the hydroxyl radical producing capability of the sample.

Human Hb that was primarily in the form of metHb was incubated in dry and hydrated (20 µg/µl) states over a 42 day period in ambient conditions. Samples were then diluted to a concentration of 1.82 µg/µl, and 16.5 µl aliquots (30 µg) were taken from each sample. Aliquots were added to 300 µl of 100 mM deoxyribose and allowed to incubate at 37°C for fifteen minutes. To each sample was added 400 µl of each 1% w/v TBA in 0.05 M NaOH and 2.8% w/v trichloroacetic acid (TAA). Samples were incubated at 95°C for 15 minutes.

Due to a number of factors, a buffer was not used for the reactions described above. First, some buffers such as Tris and Hepes are scavengers of OH•. Second, though a phosphate buffer mimics an *in vivo* situation, iron ions can bind to the buffer, to the deoxyribose, or to other components of the reaction mixture. Iron-phosphate complexes are weakly active in producing ‘free’ OH• [40]. All reactions were carried out in quadruply filtered de-ionized water.

To detect the MDA-TBA chromagen, 200 µl of the reaction assay was added to 500 µl 10% ammonium acetate buffer. The addition of the buffer allowed the pink chromagen to remain stable over the period required to conduct spectrophotometric measurements at 532 nm. The absorption was used to determine relative amounts of deoxyribose degradation by aged samples.
